# Osteoconductivity and Hydrophilicity of TiO_**2**_ Coatings on Ti Substrates Prepared by Different Oxidizing Processes

**DOI:** 10.1155/2012/495218

**Published:** 2012-12-18

**Authors:** Dai Yamamoto, Ikki Kawai, Kensuke Kuroda, Ryoichi Ichino, Masazumi Okido, Azusa Seki

**Affiliations:** ^1^Department of Materials Science and Engineering, Graduate School of Engineering, Nagoya University, Nagoya 464-8603, Japan; ^2^EcoTopia Science Institute, Nagoya University, Nagoya 464-8603, Japan; ^3^Hamri Co., Ltd., Tokyo 110-0005, Japan

## Abstract

Various techniques for forming TiO_2_ coatings on Ti have been investigated for the improvement of the osteoconductivity of Ti implants. However, it is not clear how the oxidizing process affects this osteoconductivity. In this study, TiO_2_ coatings were prepared using the following three processes: anodizing in 0.1 M H_3_PO_4_ or 0.1 M NaOH aqueous solution; thermal oxidation at 673 K for 2 h in air; and a two-step process of anodizing followed by thermal oxidation. The oxide coatings were evaluated using SEM, XRD, and XPS. The water contact angle on the TiO_2_ coatings was measured as a surface property. The osteoconductivity of these samples was evaluated by measuring the contact ratio of formed hard tissue on the implanted samples (defined as the *R*_B-I_ value) after 14 d implantation in rats' tibias. Anatase was formed by anodizing and rutile by thermal oxidation, but the difference in the TiO_2_ crystal structure did not influence the osteoconductivity. Anodized TiO_2_ coatings were hydrophilic, but thermally oxidized TiO_2_ coatings were less hydrophilic than anodized TiO_2_ coatings because they lacked in surface OH groups. The TiO_2_ coating process using anodizing without thermal oxidation gave effective improvement of the osteoconductivity of Ti samples.

## 1. Introduction

Titanium has been widely used in dental and orthopedic implants because of its good biocompatibility and high corrosion resistance [1, 2]. However, Ti in itself does not always show good performance to form hard tissue on its surface in living bodies. Therefore, proper surface treatment to enhance bone-forming ability, as represented by hydroxyapatite (HAp) coating [3–10], has been studied for a long time. Similar to HAp, TiO_2_ is also important as an osteoconductive substance because it has been shown to exhibit strong physicochemical fixation with living bone, even though it is not a component of natural bone [11]. There are many coating processes to create TiO_2_ films on Ti substrates, such as thermal oxidation [12], chemical methods [13–15], physical vapor deposition [16, 17], and anodizing [18–21]. These processes are classified into hydroprocesses and pyroprocesses. Anodizing is a widely used hydroprocess; it is performed in various aqueous solutions at an arbitrary applied voltage. On the other hand, thermal oxidation is one of pyroprocesses, which is performed in various atmospheres at high temperature. Both processes have been used to prepare bioactive TiO_2_ coatings on Ti [20, 22–25].

Previously, various parameters of TiO_2_ coating, such as crystal structure, surface roughness, and film thickness, were reported to influence the osteoconductivity of the coating [20, 27, 28]. Even though these parameters can be altered simultaneously when changing the oxidizing condition, they have not been well controlled to compare the surface properties of TiO_2_ coatings prepared with different oxidizing processes in previous works, which made it unclear what kind of surface property had influence on the hard tissue formation in *in vivo* test. Therefore, in this study, we prepared TiO_2_ coatings with controlled surface structure on Ti using anodizing and/or thermal oxidation and investigated the chemical influence of oxidizing processes on the osteoconductivity.

## 2. Materials and Methods

### 2.1. Preparation of Ti Substrates

Commercially pure Ti (Cp-Ti) disks (for anodizing, area = 1.13 cm^2^) and plates (for thermal oxidation, area = 1 cm^2^) were used as substrates to prepare coatings for surface analysis, and rods (for both anodizing and thermal oxidation, dimensions = *ϕ*2 × 5 mm) for *in vivo *testing. The Cp-Ti disks were covered with epoxy resin, except for the face that would be in contact with the aqueous solution. All of the substrates were polished by emery paper followed by buffing using Al_2_O_3_ particles (particle size = 0.05 *μ*m). After polishing, the substrates were cleaned and then degreased with ethanol.

### 2.2. TiO_2_ Coatings

 The following three methods were used to form TiO_2_ coatings on Ti substrates. The preparation conditions were selected in each case so that all of the oxide coatings had the same film thickness to exclude the influence of that variable on the osteoconductivity of the formed coatings.

#### 2.2.1. Anodizing in Aqueous Solutions

A Ti substrate and a Pt coil were set as anode and cathode, respectively, and reference electrode was not used. The anodizing voltage was increased from 0 V up to 100 V in 0.1 M H_3_PO_4_ aqueous solution and up to 80 V in 0.1 M NaOH aqueous solution at a rate of 0.1 V s^−1^. The aqueous solution was stirred and kept at a constant temperature (298 K) in a water bath during anodizing. This oxidation process is denoted as “AZ treatment” in the following description.

#### 2.2.2. Thermal Oxidation

Titanium substrates were heated to 673 K at a rate of 4 K min^−1^ in air in an electric resistance furnace, and kept at that temperature for 2 h. The substrates were then cooled in the furnace, in the same atmosphere. This oxidation process is denoted as “TO treatment.”

#### 2.2.3. Two-Step Process of Anodizing and Thermal Oxidation

 In the first step, substrates were anodized with the voltage increasing from 0 V to 70 V in 0.1 M H_3_PO_4_ aqueous solution or up to 50 V in 0.1 M NaOH aqueous solutions at a rate of 0.1 V s^−1^. The anodized samples then were heated to 673 K at a rate of 4 K min^−1^ in air in an electric resistance furnace, and kept at that temperature for 2 h. The substrates were then cooled in the furnace, in the same atmosphere. This two-step oxidation process is denoted as “AZ-TO treatment.”

### 2.3. Analysis of the Coatings

All of the samples were sterilized using an autoclave unit at 394 K for a period of 20 min before following. The surface morphology was observed using a scanning electron microscope (SEM). The coated films were identified using thin-film X-ray diffraction (XRD) and X-ray photoelectron spectroscopy (XPS). The surface roughness was measured using a confocal laser scanning microscope with an analysis area of 150 *μ*m × 112 *μ*m. The arithmetical mean of the surface roughness (Ra) was used, as this value was not distorted by any local scarring of the sample. The contact angle of distilled water (WCA) was measured at three different points for each sample using a 2 *μ*L droplet of distilled water 24 h after autoclaving, and the average value was used as the WCA value.

### 2.4. *In Vivo* Test

*In vivo* tests were performed for all of the AZ-treated, TO-treated, AZ-TO-treated, and as-polished samples. Because the experimental procedure for our *in vivo* study was almost the same as described in previous reports [8], it is described only briefly here. Before surgery, all of the implants were cleaned in distilled water and immersed in a chlorhexidine gluconate solution. Ten-week-old male Sprague Dawley rats (Charles River Japan, Inc., Japan) were used in our experimental procedures. The samples were implanted in the tibial metaphysis of the rats. A slightly oversized hole, which did not pass through to the rear side of the bone, was created using a low-speed rotary drill. Subsequently, the implants were inserted into these holes, and then the subcutaneous tissue and skin were closed and sterilized.

The rats were sacrificed after a period of 14 d, and the implants with their surrounding tissue were retrieved. The samples were fixed in a 10% neutral buffered formalin solution, dehydrated in a graded series of ethanol, and embedded in methylmethacrylate. Following polymerization, each implant block was sectioned longitudinally into 20 *μ*m thick slices. These sections were then stained with toluidine blue.

The sum of the linear bone contact with the implant surface was measured and was expressed as a percentage over the entire implant length (the bone-implant contact ratio, *R*_B-I_) in the cancellous bone and the cortical bone parts [8]. Significant differences in the bone-implant contact ratio were analyzed statistically using the Tukey-Kramer method [29]. Differences were considered statistically significant at the *P* < 0.05 level. This animal study was conducted in a laboratory accredited by AAALAC International (Association for Assessment and Accreditation of Laboratory Animal Care International). 

## 3. Results and Discussion

### 3.1. Evaluation of Oxide Coatings

**Figure 1:**
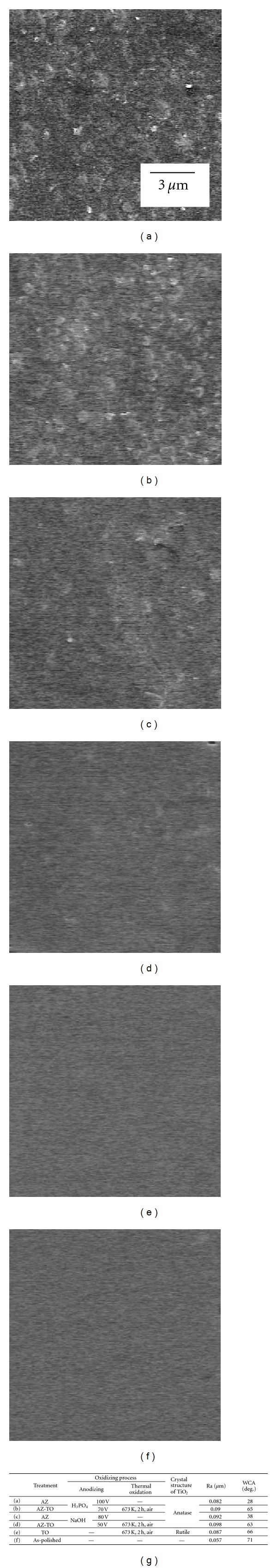
The surface SEM images, surface roughness Ra, and WCA of the samples prepared in this study.

**Figure 2:**
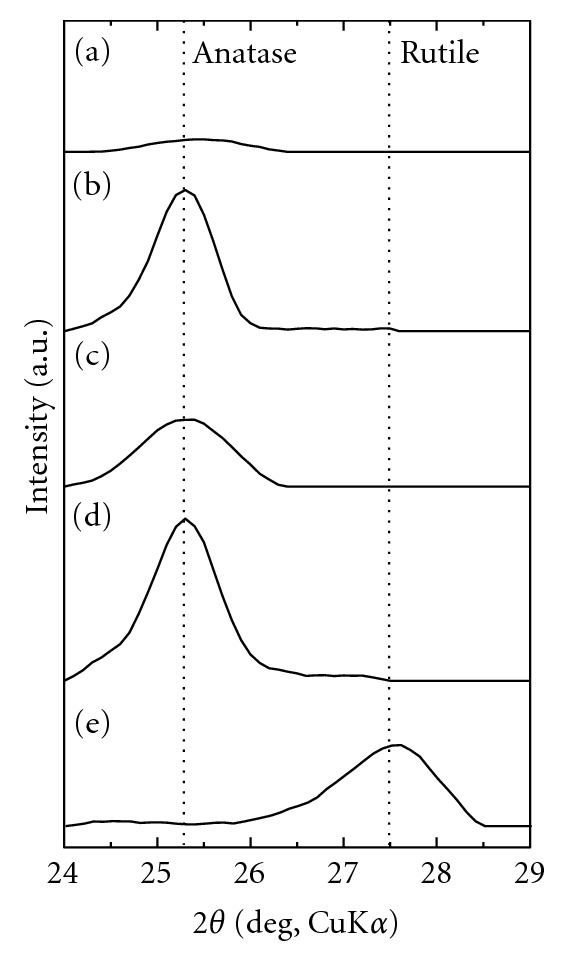
The XRD patterns of the surface coatings on samples treated by (a) AZ treatment up to 100 V in 0.1 M H_3_PO_4_ aq., (b) AZ-TO treatment (anodized in H_3_PO_4_ aq.), (c) AZ treatment up to 80 V in 0.1 M NaOH aq., (d) AZ-TO treatment (anodized in NaOH aq.), and (e) TO treatment.

**Figure 3:**
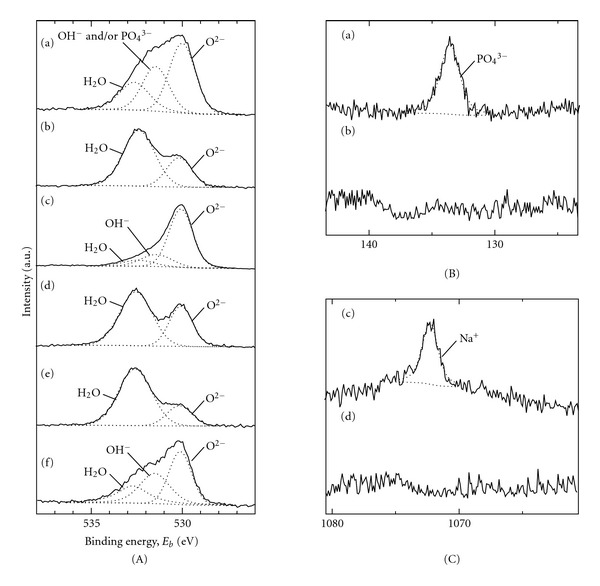
The (A) O1s, (B) P2p, and (C) Na1s XPS surface spectra of samples treated by (a) AZ treatment up 100 V in 0.1 M H_3_PO_4_ aq., (b) AZ-TO treatment (anodized in H_3_PO_4_ aq.), (c) AZ treatment up to 80 V in 0.1 M NaOH aq., (d) AZ-TO treatment (anodized in NaOH aq.), and (e) TO treatment.

 The surface morphology and surface roughness of these samples are shown in Figure 1.  Figure 2 shows the XRD patterns of the samples after AZ treatment in (a) 0.1 M H_3_PO_4_ aqueous solution up to 100 V, (b) 0.1 M NaOH aqueous solution up to 80 V, (c) TO treatment at 673 K for 2 h in air, and AZ-TO treatment (anodized in (d) H_3_PO_4_ and (e) NaOH aqueous solution).  Figure 3 shows the XPS spectra ((A) O1s, (B) P2p, and (C) Na1s) of each of the samples. 

Surface was kept fine (Ra/*μ*m < 0.1) after any oxidizing process (Figures 1(a)–1(e)). All of the formed oxide coating had the same film thickness of *c.a.* 120 nm [30]. However, the crystal structure of the oxide coating was different depending on the oxidizing process; anatase-type TiO_2_ was formed by AZ treatment (Figures 2(a), and 2(b)), but rutile-type TiO_2_ by TO treatment (Figure 2(e)). Each of AZ-treated samples contained PO_4_^3−^ or Na^+^ in the oxide coating which derived from aqueous solutions (Figures 3(B)(a) and 3(B)(c)), as described in our previous report [27]. Anatase was also formed by AZ-TO treatment, which was the same crystal structure as the AZ-treated coatings (Figures 2(b), and 2(d)), and no rutile was detected in the coatings using XRD, despite the thermal oxidation.

 The XPS analysis showed different O1s spectra depending on the oxidation process. When deconvoluting the O1s spectrum of the as-polished sample in the same way as in a previous work [31], the spectrum was divided into three predominant peaks (530.1, 531.5, and 532.5 eV) originating from O^2-^, OH group, and adsorbed water (Figure 3(A)(f)), which derived from the thin natural oxide layer on the Ti substrate. These components were also present after anodizing (Figures 3(A)(a), and 3(A)(c)). However, OH groups was lost from the surface after thermal oxidation (Figures 3(A)(b), 3(A)(d), and 3(A)(e)), in the same way as a previous report by Zhao et al. [24]. Instead, large amounts of water molecule adsorbed on the surface of thermally oxidized samples. The absence of PO_4_^3−^ or Na^+^ on the AZ-TO-treated samples (Figures 3(B)(b) and 3(B)(d)), which was detected on the anodized-only samples (Figures 3(B)(a) and 3(C)(c)), supports the idea that the oxide layer grows outward during thermal oxidation, as noted by Kumar et al. [32]. The sample anodized in H_3_PO_4_ aqueous solution is considered to have surface OH groups, but the corresponding peak could not be separated from the peak of PO_4_^3−^ at the binding energy of around 531.5 eV (Figure 3(A)(a)). Although it is not shown in this figure, hydrocarbon was detected as a contaminant at the surface of all of the samples at the same adsorption level.

 The surface hydrophilicity of oxidized samples was different depending on their oxidizing processes. As listed in Figure 1, WCA values decreased largely from 71 deg. (f) to less than 40 deg. ((a), (c)) after AZ treatment, regardless of the type of aqueous solutions used for anodizing. The anodizing process did not significantly change the amounts of OH group and adsorbed hydrocarbon at the surface. It was already shown that solute ions (PO_4_^3−^ and Na^+^) contained in anodized coatings did not contribute to the reduction of WCA [28]. On the other hand, all of the thermally oxidized samples (i.e., TO-treated sample (Figure 1(e)) and AZ-TO-treated samples (Figures 1(b), and 1(d)) had high WCA values similar to the as-polished sample. Since TO-treated sample and AZ-TO-treated samples had almost the same WCA values in spite of different crystal structure of formed TiO_2_, it is thought that the crystal structure of TiO_2_, whether anatase or rutile, did not have strong influence on the hydrophilicity. In addition, the increased amount of adsorbed water molecule on thermally treated samples did not contribute to the decrease of the WCA value. Based on these results, it is reasonable to think that the absence of OH groups on the surface is responsible for the high WCA values of TO- and AZ-TO-treated samples. In other words, anodizing can improve the hydrophilicity of as-polished samples as a consequence of forming TiO_2_ films having surface OH groups, but thermal oxidation does not improve the hydrophilicity because of the lack of surface OH groups on the TiO_2_ films.

### 3.2. **In Vivo ** Evaluation

**Figure 4:**
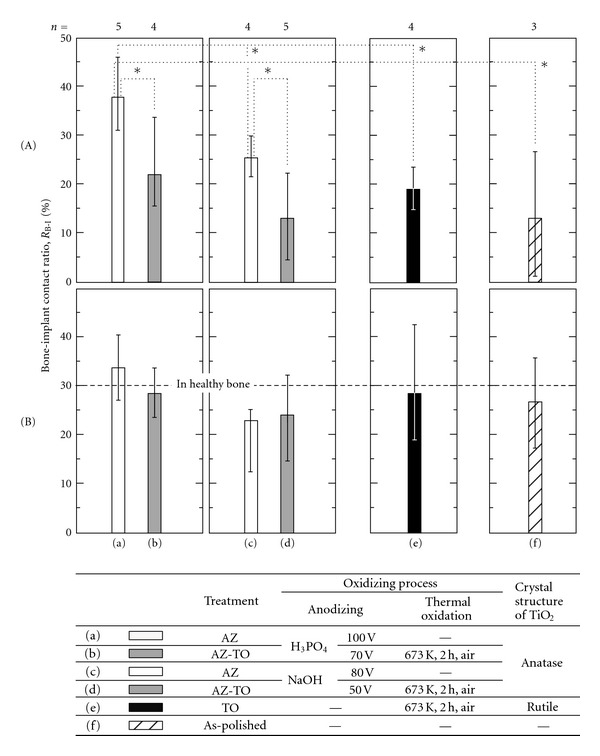
Bone-implant contact ratio, *R*_B-I_, of Ti samples in both (A) the cortical bone part and (B) the cancellous bone part. *P < 0.05.

Figure 4 shows the bone-implant contact ratios, *R*_B-I_, of the samples in both the cortical bone part (A) and the cancellous bone part (B). In the cancellous bone part, the *R*_B-I_ values approached nearly 30% with any coating process; this value agreed well with the percentage of hard tissue in healthy cancellous bone in rats. According to this, it is difficult to judge the influence of the oxidizing process on *R*_B-I_ values in cancellous bone parts in the early stages after implantation. On the other hand, in the cortical bone part (A), the *R*_B-I_ values were obviously different depending on the oxidizing process for the slow formation rate of hard tissue. Therefore, we will focus on the cortical bone part in the following discussion.

**Figure 5:**
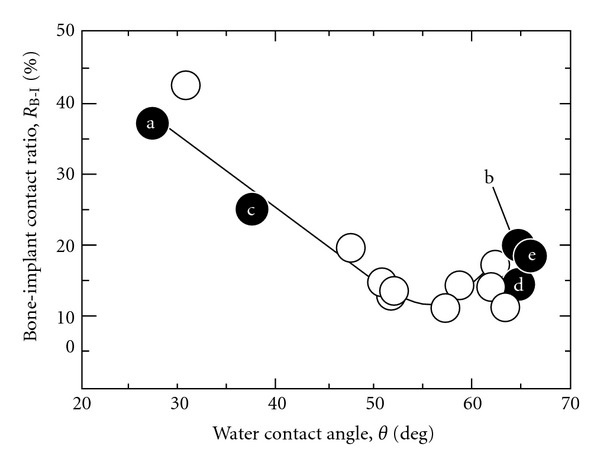
Relationship between the WCA and *R*_B-I_ values in the cortical bone part. The symbols in closed circle correspond to the letters in Figure 4. Open circles represent the data for the sample anodized in various aqueous solutions, taken from previous work [26].

 The *R*_B-I_ values of samples varied significantly depending on the oxidizing process. Both AZ-treated samples (Figures 4(A)(a) and 4(A)(c)) tended to have higher *R*_B-I_ values than the as-polished sample (Figure 4(A)(f)). In contrast, the *R*_B-I_ values of thermally oxidized samples (b), (d), (e) were as low as that of the as-polished sample. The low *R*_B-I_ values of the AZ-TO-treated samples mean that the *R*_B-I_ values of the AZ-treated samples decreased significantly after additional thermal oxidation, regardless of the type of aqueous solutions used for AZ treatment (Figures 4(A)(b), and 4(A)(d)). These results indicate that thermal oxidation attributed to decrease the osteoconductivity of TiO_2_ coatings, and also that the different crystal structures of TiO_2_ did not influence the osteoconductivity. Furthermore, all of the thermally oxidized samples were similar, with WCA values ranging from 60 (deg.) to 70 (deg.) and also *R*_B-I_ values ranging from 10% to 20%. These values followed the relation between *R*_B-I_ and WCA reported in our previous study (Figure 5) [28]. This means that variation of surface hydrophilicity was the reason for the different osteoconductivity of TiO_2_ coatings prepared with different oxidizing process. Therefore, it was thought that the surface properties, such as WCA and the amount of OH groups, strongly affected the osteoconductivity, rather than the coating substance, crystal structure, and coating process. TiO_2_ coating processes using only anodizing and not thermal oxidation improved the osteoconductivity of Ti samples effectively.

## 4. Conclusions

 In this study, we prepared TiO_2_ coatings with controlled surface structure on Ti using anodizing and/or thermal oxidation and investigated the chemical influence of oxidizing processes on the osteoconductivity. The following results were obtained.

1. Anatase was formed by anodizing, and rutile by thermal oxidation, but the difference of TiO_2_ crystal structure did not influence their osteoconductivity.
2. Anodized TiO_2_ coatings were hydrophilic, but thermally oxidized TiO_2_ coatings were less hydrophilic than anodized-only TiO_2_ coatings because of the lack of surface OH groups.
3. The TiO_2_ coating process using only anodizing, not including thermal oxidation, gave the effective improvement of the osteoconductivity of Ti samples.
